# Transient increase in skeletal-related events after discontinuation of high-dose denosumab in cancer patients

**DOI:** 10.1016/j.jbo.2025.100717

**Published:** 2025-10-11

**Authors:** Nokitaka Setsu, Nobuhiko Yokoyama, Taito Esaki, Masafumi Yamaguchi, Eriko Tokunaga, Takahito Negishi

**Affiliations:** a Department of Orthopedic Surgery, NHO Kyushu Cancer Center, 3-1-1 Notame, Minami-ku, Fukuoka, Japan; b Department of Gastrointestinal and Medical Oncology, NHO Kyushu Cancer Center, 3-1-1 Notame, Minami-ku, Fukuoka, Japan; c Department of Thoracic Oncology, NHO Kyushu Cancer Center, 3-1-1 Notame, Minami-ku, Fukuoka, Japan; d Department of Breast Oncology, NHO Kyushu Cancer Center, 3-1-1 Notame, Minami-ku, Fukuoka, Japan; e Department of Urology, NHO Kyushu Cancer Center, 3-1-1 Notame, Minami-ku, Fukuoka, Japan

**Keywords:** Denosumab, Bone metastases, SRE, Discontinuation, Rebound, ALP

## Abstract

**Background:**

Denosumab is widely used to prevent skeletal-related events (SREs) in patients with bone metastases. However, rebound bone resorption after discontinuation is recognized. In osteoporosis, discontinuation of low-dose denosumab increases multiple vertebral fractures, but data on high-dose discontinuation remain limited.

**Methods:**

Among 493 patients treated with high-dose denosumab at our institution (2014–2023), 78 met eligibility criteria. SREs during and after treatment were compared using each patient as their own control. SREs were defined as pathological fracture, spinal cord compression, or radiotherapy/surgery for metastatic bone pain. Hypercalcemia, benign fragility fractures, and serum ALP level were also assessed.

**Results:**

A total of 11 SREs were observed during denosumab and 24 after discontinuation. Post-discontinuation incidence was 14.1 per 1,000 person-months, 3.3 times higher than during treatment (95 % CI, 1.4–7.8). The increase was significant at 6–15 months, peaking at 12–15 months (IRR 8.7, 95 % CI, 2.8–27.5), and declined thereafter. Fewer denosumab doses were also associated with a higher risk of SREs after discontinuation. Two benign fragility fractures occurred during denosumab and four after discontinuation. Grade ≥ 3 hypercalcemia occurred only after discontinuation (3 cases). Transient ALP elevation at 9–18 months was observed in patients with post-discontinuation SREs, and ALP ≥ 95 U/L at 6 months predicted subsequent SREs (AUC 0.80).

**Conclusion:**

SREs increased significantly 6–15 months after high-dose denosumab discontinuation. Elevated ALP was associated with post-discontinuation SREs. These findings emphasize that discontinuation contributes to SRE risk, possibly via rebound bone resorption, and underscore the importance of continuation of therapy whenever possible.

## Highlights

• SREs increased significantly 6–15 months after denosumab discontinuation.
• SRE increase was transient, suggesting possible involvement of rebound bone resorption.
• ALP was also transiently elevated, with ALP at 6 months predicting later SREs.

## Graphical abstract

**Figure:**
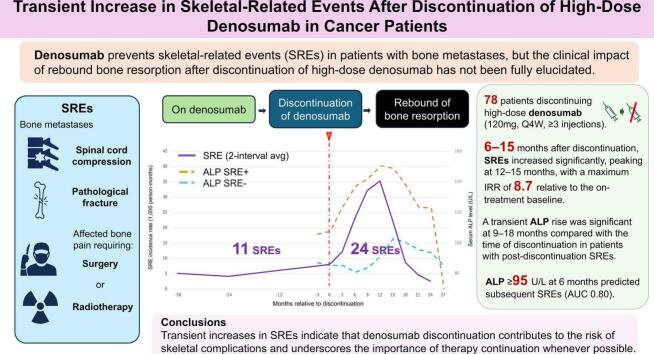

## 1 Introduction

Bone metastases from solid tumors and bone lesions of hematologic malignancies are frequently accompanied by skeletal-related events (SREs), defined as spinal cord compression, pathological fracture, or the need for surgery or radiotherapy due to metastatic bone pain [[1], [2], [3]]. SREs are associated with loss of mobility and social functioning, impaired quality of life (QoL), increased health care costs, and reduced survival [4,5].

Osteoclast activation has been reported to contribute to the pathophysiology of bone metastases [6]. Denosumab, a monoclonal antibody targeting RANKL, inhibits osteoclast differentiation and reduces bone resorption. Based on this mechanism, low-dose denosumab (60 mg every 6 months) is approved for osteoporosis, while high-dose denosumab (120 mg every 4 weeks) is approved for bone metastases from solid tumors and bone lesions of multiple myeloma [7]. Zoledronic acid, a bisphosphonate incorporated into osteoclasts that induces apoptosis, has also long been used in this setting. Together with denosumab, these drugs are categorized as anti-resorptive agents or bone-targeting agents (BTAs). According to the European Society for Medical Oncology (ESMO) guidelines [1], BTAs should be continued indefinitely, including in the hospice setting. However, treatment may be withheld in selected patients with favorable prognostic features such as oligometastatic disease, a low perceived risk of skeletal complications, and durable responses to systemic therapy [1].

High-dose denosumab has been reported to provide modestly better prevention of SREs compared with zoledronic acid [3,7,8], as well as improved pain control [9,10]. In contrast, discontinuation of denosumab may increase the risk of SREs, not only due to loss of efficacy but also because of rebound activation of bone resorption. A rebound phenomenon characterized by bone resorption exceeding pretreatment levels has been described after denosumab discontinuation [11]. In osteoporosis, discontinuation of low-dose denosumab results in rapid bone loss and an increased incidence of multiple vertebral fractures compared with untreated patients [12,13]. During denosumab therapy, accumulation of osteoclast precursors [14] and osteoclast fission to osteomorph [15] have been reported, which may create a biological environment that facilitates reactivation of osteoclast activity after discontinuation. Osteomorphs are recently identified circulating cells that emerge when osteoclasts fragment after bone resorption and can subsequently re-fuse to form active osteoclasts again. This reversible cellular state is thought to play a key role in bone remodeling and may help explain the rebound increase in bone resorption observed after denosumab discontinuation [15].

Although an increase in SREs after discontinuation of high-dose denosumab is considered an expected phenomenon, only a limited number of studies have investigated this issue [16]. Jacobson et al. followed 1,414 patients for one year after denosumab discontinuation and, using machine learning with real-world data, identified prior SREs and shorter treatment duration as primary risk factors. However, their study did not clarify the extent to which discontinuation itself contributes to an increased risk of SREs.

Hypercalcemia after discontinuation of high-dose denosumab has been frequently reported in children with high bone turnover [17,18]. Sporadic cases, including those with bone metastases, have also been described in adults [19,20]. In a literature review of 49 cases, Horiuchi et al. found that hypercalcemia was more common in males before epiphyseal closure and in females among adults, and that younger age was associated with earlier onset of hypercalcemia after discontinuation. They concluded that high baseline bone turnover was a risk factor for hypercalcemia [21]. Nevertheless, the risk and incidence of hypercalcemia after discontinuation of high-dose denosumab remain unclear.

In a cohort of 30 lung cancer patients, three cases of non-cancerous vertebral fractures were observed after discontinuation of high-dose denosumab [22]. Similar to the findings in osteoporosis treated with low-dose denosumab, benign fragility fractures may also occur after discontinuation of high-dose denosumab. However, the characteristics and frequency of benign fragility fractures in cancer patients have not been well defined.

This study aimed to evaluate the risk of increased skeletal-related events after discontinuation of high-dose denosumab and to investigate their clinical characteristics in a single-center retrospective analysis. Several studies have reported that elevated alkaline phosphatase (ALP) is a risk factor for SREs in patients with bone metastases [[23], [24], [25]], suggesting that ALP may serve as a surrogate marker of bone turnover. Therefore, we examined ALP, along with hypercalcemia and benign fragility fractures, in addition to SREs..

## 2 Patients and methods

To examine the association between discontinuation of high-dose denosumab (120 mg every 4 weeks) and the incidence of skeletal-related events (SREs), we conducted a self-controlled analysis in which each patient served as their own control, comparing SREs during treatment and after discontinuation.

### 2.1 Patient selection

We identified 493 patients who had received high-dose denosumab between January 2014 and January 2023 from electronic medical records. Patients were identified in June 2023, and follow-up was completed in June 2025. Of these, 69 patients who continued treatment were excluded, leaving 424 patients who were discontinued. We further excluded 315 patients whose follow-up was < 3 months after discontinuation, and 31 patients who had received < 3 doses. The final cohort comprised 78 patients, who were observed until death, end of follow-up, restart of denosumab, or the end of the study (June 30, 2025). In one patient who restarted denosumab during follow-up, observation was censored at the time of retreatment. Patients who had received zoledronic acid or other bisphosphonates before denosumab initiation or after its discontinuation were not excluded and were allowed in this study. Orthopedic surgeons (NS and NY) reviewed medical records and imaging.

### 2.2 Outcome definitions

SREs were defined as metastatic pathologic fractures, spinal cord compression, or bone pain from metastases requiring radiotherapy or surgery. Multiple SREs in the same patient were recorded, but repeated radiotherapy to the same site was counted only at the initiation of the first course. Fractures were confirmed by medical records or imaging, and patient-reported fractures treated at outside institutions were also included. Grade ≥ 3 hypercalcemia (graded by CTCAE v5.0) and all-grade hypercalcemia were analyzed separately. G ≥ 3 hypercalcemia was not counted when attributable to other obvious causes such as terminal illness, deterioration of general condition, or dehydration. All-grade hypercalcemia was assessed for its frequency and potential as a predictor of subsequent SREs. For all-grade hypercalcemia, only the first event before and after discontinuation was recorded. Benign fragility fractures, which were judged by an orthopedic surgeon’s imaging review to be unrelated to bone metastases, were counted. To evaluate the impact of discontinuation, we examined whether SREs occurred in association with progression of the underlying malignancy. Non-skeletal progression was defined as radiographic evidence of progression outside bone, rising tumor markers judged as indicative of progression, or worsened clinical symptoms attributable to tumor progression. Patients without relevant imaging or tumor marker data were classified as unevaluable.

### 2.3 Serum alkaline phosphatase level

Serum alkaline phosphatase (ALP) was assessed retrospectively at the discontinuation date (±2 weeks), before 3 months, and every 3 months thereafter up to 27 months (±4 weeks), and at the time of an SRE. When ALP was not measured exactly on the target date, the value obtained closest to that date within the specified window was used. If ALP was not measured on the exact day of the SRE, values obtained within the preceding 4 weeks were used. ALP values influenced by non-skeletal causes, such as concomitant elevation of AST/ALT ≥ grade 1, suspected biliary obstruction, γGTP ≥ grade 3, or marked increases (Grade ≥ 3 or ≥ 2-fold increase within 3 months) within 3 months before death, were excluded. The missing values were left as missing. Until December 2020, serum ALP was measured using the Japanese Society of Clinical Chemistry (JSCC) method. Values were converted to the International Federation of Clinical Chemistry and Laboratory Medicine (IFCC) method by multiplying by 0.35, as previously reported [26].

### 2.4 Calculation of incidence rates

Incidence rates were calculated per person-month, using the number of person-days divided by 30.44 as the denominator. The denosumab treatment period was defined as the number of days between the first and last injection plus 28 days, based on the previous study of denosumab discontinuation [22], the standard dosing interval of 28 days, and the mean terminal half-life of denosumab of 26 days [27]. The mean dosing interval was calculated by dividing the denosumab treatment period by the number of doses. Treatment gaps > 6 months were excluded from the treatment period, and SREs occurring during such gaps were not included in the analysis. To estimate the incidence rate during treatment, SREs that occurred on the day of treatment initiation, for which the relationship to treatment could not be determined, or SREs that directly led to treatment discontinuation were excluded. The period after 28 days from the last administration was defined as the discontinuation period, and events occurring thereafter were defined as post-discontinuation events.

### 2.5 Statistical analysis

For comparison of event trends before and after discontinuation, the observation period was divided into predefined intervals, and incidence rates per person-month were calculated for each. Ninety-five percent confidence intervals (CIs) for incidence rates were calculated assuming a Poisson distribution, including recurrent events per patient. When no events occurred, only the upper limit of the 95 % CI was estimated using the Poisson distribution. Comparisons between pre- and post-discontinuation periods were made using incidence rate ratios (IRRs) with 95 % CIs, also based on the Poisson distribution. To confirm the robustness of the primary Poisson regression, SRE incidence was re-evaluated by Fine & Gray competing-risk analysis with death as a competing event and by a Poisson generalized estimating equation (GEE) model with an exchangeable correlation structure, using patient ID as the cluster, period (on– vs. post-discontinuation) as the exposure, and person-days as an offset; robust standard errors addressed overdispersion. These sensitivity analyses excluded the cases with SREs that occurred on the day of treatment initiation or SREs that directly led to treatment discontinuation, similar to baseline incidence speculation. For paired comparisons of the number of all-grade hypercalcemia events before and after discontinuation, the McNemar test was used. Fisher’s exact test was applied to other contingency tables. The Mann–Whitney *U* test was used for continuous variables between independent groups, and the Wilcoxon signed-rank test was used for paired continuous variables. Statistical significance was defined as P < 0.05. Analyses were conducted using EZR [28].

### 2.6 Ethics

This study was approved by the Institutional Review Board of the authors’ institution. The study was conducted in accordance with the Declaration of Helsinki.

## 3 Results

### 3.1 Patient characteristics

A total of 78 patients were included in the analysis: 32 men and 46 women, aged 36–84 years (mean, 66.7 years). The primary tumors were lung cancer in 32 patients (including 2 with multiple cancers, but bone metastases were attributed to lung cancer), breast cancer in 18, prostate cancer in 12, renal cell carcinoma in 6, multiple myeloma in 3, and other cancers in 7. In one patient who restarted denosumab 32 months after discontinuation, observation was censored at retreatment. The median follow-up was 365 days (mean, 692 days). The median overall survival was 601 days (Supplementary figure 1). Data on the study endpoint were available for all 78 patients.

Reasons for discontinuation were oral conditions in 32/78 patients (including 17/78 with osteonecrosis of the jaw), unknown in 28/78, patient preference in 5/78, transfer to palliative care in 2/78, hypocalcemia in 2/78, disease progression in 1/78, and other reasons in 8/78. Three patients had treatment interruptions > 6 months due to tooth extraction, gingival bleeding, and an unknown reason. Excluding these intervals, the denosumab treatment duration ranged from 11.9 to 417.6 weeks (mean, 95.9 weeks). The number of doses ranged from 3 to 82 (mean, 20.1). The mean dosing interval was 5.0 weeks (range, 4.0–17.9 weeks). Only two patients had dosing intervals > 7 weeks: one with a planned prolongation of the dosing interval (mean, 9.1 weeks) and one with unexplained prolongation (mean, 17.9 weeks).

### 3.2 Incidence of SREs during and after denosumab treatment

During denosumab treatment, 11 patients experienced 11 SREs. The mean time from treatment initiation to SRE was 6.4 months (range, 0–22.7 months). Five patients developed SREs within 3 months of initiation, including 2 who developed SREs on the first day of administration. These 2 cases were excluded from the calculation of incidence during treatment. Four patients developed SREs within 28 days of the final injection, including 2 on the same day. These events were considered the direct cause of discontinuation. To estimate the incidence of SREs during treatment as accurately as possible, cases with SREs on the final injection day were included, whereas 2 cases with SREs after discontinuation were excluded. After excluding these 4 cases, 7 SREs occurred during 1,659.7 person-months, corresponding to an incidence rate of 4.2 per 1000 person-months. These 4 cases were also excluded from the sensitivity analyses described below.

**Fig. 1:**
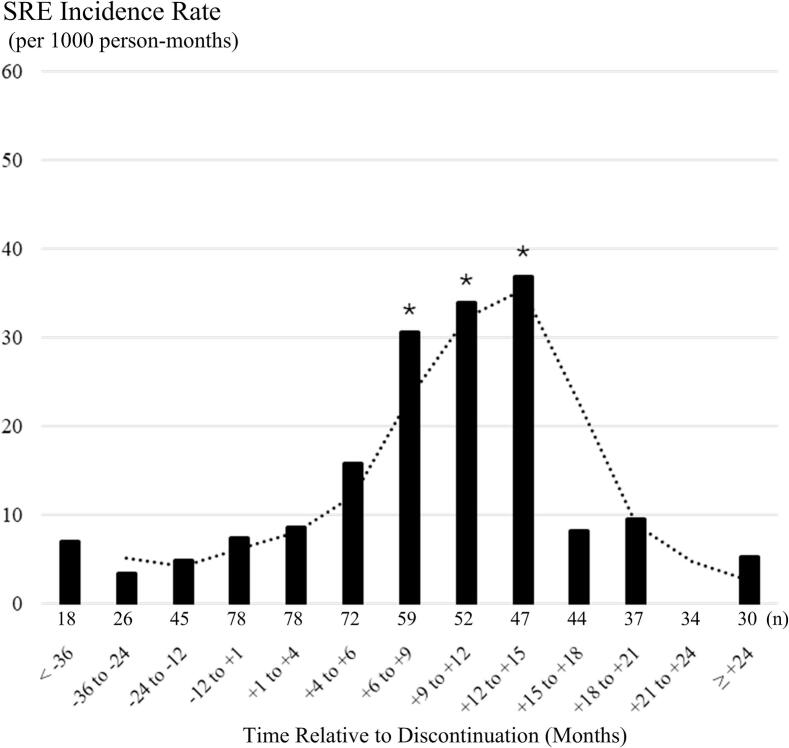
Incidence of skeletal-related events (SREs) around the discontinuation of denosumab. The dashed line illustrates the trend using a two-period moving average of the incidence rate. n indicates the number of patients at risk. For the on-treatment period, n refers to the number of patients remaining at the end of each interval; for the post-discontinuation period, it refers to the number at the beginning of each interval. Asterisks indicate a significant increase compared with the pre-discontinuation baseline rate (IRR > 1 with the 95 % CI lower bound > 1, based on a Poisson regression model).

After discontinuation, 24 SREs occurred in 18/78 patients, including 2 who had also developed SREs during treatment. No patient experienced more than 2 SREs. The temporal pattern of incidence is shown in Fig. 1a. The incidence after discontinuation was 14.1 per 1000 person-months, 3.3 times higher than during treatment (IRR 3.3, 95 % CI 1.4–7.8). Incidence was significantly higher at 6–9 months (IRR 5.79, 95 % CI 1.7–19.8), 9–12 months (IRR 8.0, 95 % CI 2.6–25.3), and 12–15 months (IRR 8.7, 95 % CI 2.8–27.5), but not at other time intervals.

Cumulative incidence curves for first SREs during denosumab treatment and after discontinuation were calculated with Fine and Gray’s competing-risk method with death as a competing event (Supplementary Fig. 2). For the on-treatment period, time was measured from the first denosumab injection until either the first SRE or 28 days after the last injection, whichever came first. During treatment, the cumulative incidence was 5.7 % (95 % CI, 2.2–14.5) at 6 months, 7.5 % (3.2–17.3) at 12 months, 10.1 % (4.5–21.7) at 18 months, and 13.6 % (6.3–27.8) at 24 months. After discontinuation, it was 4.1 % (1.1–10.4) at 6 months, 15.0 % (7.9–24.2) at 12 months, 19.1 % (11.0–29.0) at 18 months, and 20.5 % (12.1–30.5) at 24 months. Although the 6-month incidence was higher during treatment, the post-discontinuation curve surpassed the on-treatment curve by 12 months (P = 0.226).

In the paired before–and–after analysis excluding four patients who were described above (n = 74), a GEE Poisson model with log(person-time) as an offset yielded an incidence rate ratio (IRR) of 3.5 (95 % CI 1.4–8.7; p = 0.006). The within-patient correlation was negligible (α = 0.02, p = 0.89), and robust (sandwich) standard errors accounted for modest overdispersion (scale = 8.82). These findings support the validity of our primary Poisson regression analyses based on the Poisson distribution assumption.

### 3.3 Characteristics of SREs

Table 1 summarizes the comparison of SREs during and after treatment. After discontinuation, metastatic pathological fractures (OR 6.25, 95 % CI 1.1–35.2, P = 0.035) and SREs in the spine (OR 8.0, 95 % CI 1.5–42.5, P = 0.012) were significantly more frequent. SREs without concomitant progression of non-skeletal disease showed a non-significant trend toward being more common after discontinuation (OR 2.33, 95 % CI 0.5–11.2, P = 0.657). When limited to events occurring more than 6 months after discontinuation, this trend was stronger (OR 5.0, 95 % CI 0.5–49.0, P = 0.193).

**Table 1:** Comparison of SREs during denosumab treatment and after discontinuation.

Category	SREs during denosumab (n)	SREs after discontinuation (n)	Odds ratio	P value
Total SREs	11	24		
Progression status				
Non-skeletal progression absent	2	6	2.33	0.657
Non-skeletal progression present	7	9		
Not evaluable	2	9		
Event and treatment (with overlap)				
Pathological fracture	2	14	6.25	0.035*
Spinal cord compression	1	0	0	0.314
Radiation	7	14	0.80	1.000
Surgery	2	2	0.41	0.575
Site of SRE				
Spine	3	18	8.00	0.0115*
Long bones	3	3	1.67	1.000
Flat bones	4	3	1.00	1.000
Short bones	1	0	0	1.000
ALP (mean ± SD, U/L)	138.4 ± 72.2	182.1 ± 94.6		0.168

Statistically significant results (P < 0.05) are indicated with an asterisk. Event and treatment categories are not mutually exclusive. SRE, skeletal-related event; ALP, alkaline phosphatase.

Table 2 compares patients with and without SREs after discontinuation. The number of denosumab doses was significantly lower in patients with SREs (14.7 ± 15.7) than in those without (21.7 ± 18.9, P = 0.0487). Treatment duration was shorter in patients with SREs (68.7 ± 72.5 weeks) than in those without (103.5 ± 91.5 weeks), although not statistically significant (P = 0.073). SREs before denosumab were observed in 30 patients. The frequency was slightly higher in patients who later developed SREs after discontinuation (44.4 % vs. 36.7 %), but not significant. SREs during treatment were observed in 11 patients (15 %) among those who later developed SREs after discontinuation, slightly more frequent in those without post-discontinuation SREs (15.0 % vs. 11.1 %), also not significant. All-grade hypercalcemia after discontinuation was more frequent in patients who developed SREs (55.6 %) than in those who did not (36.7 %). During treatment, the reverse trend was observed (11.1 % vs. 20.0 %); neither difference was significant. Eight patients switched to bisphosphonates (BPs), including 2 who received oral agents at osteoporosis doses; 3 developed SREs after discontinuation. Two patients with and two without SREs had received zoledronic acid prior to denosumab. One patient without SREs received a single dose of zoledronic acid for post-discontinuation hypercalcemia. No significant association was observed between BP therapy and SRE occurrence.

**Table 2:** Comparison of patients with and without post-discontinuation SREs.

Variable	No SRE after discontinuation (n = 60)	SRE after discontinuation (n = 18)	P-value
Demographics			
Sex (M:F)	25:35	7:11	1.000
Age, years	66.0 ± 10.9	69.2 ± 10.7	0.219
BMI, kg/m^2^	22.3 ± 4.5	23.8 ± 5.8	0.250
Primary cancers			
Lung	25	7	0.831
Breast/Prostate	22	8	
Other types	13	3	
Systemic therapy status*			
Continued same therapy	36	11	0.590
No systemic therapy	6	0	
Changed therapy	16	5	
Therapy discontinued	4	2	
Baseline SREs			
Pre-denosumab SRE	22	8	0.589
On-denosumab SRE	9	2	1.000
Denosumab exposure			
Treatment duration (weeks)	103.5 ± 91.5	68.7 ± 72.5	0.0733
Number of doses	21.7 ± 18.9	14.7 ± 15.7	0.0487†
Other outcomes			
Fragility fractures	2	3	0.0775
Hypercalcemia grade 3	1	2	0.131
Hypercalcemia grade ≥ 1	22	10	0.179
On-denosumab hypercalcemia	12	2	0.501
Bisphosphonate use			
Switch to high-dose BP	4	2	0.617‡
Oral low-dose BP	1	1	—
Prior high-dose BP	2	2	—
BP for hypercalcemia	1	0	—

Continuous variables are presented as mean ± standard deviation. SRE: skeletal-related event, BP: bisphosphonate. *Course of anti-cancer drug therapy within 3 months after denosumab discontinuation. †Statistically significant (P < 0.05). ‡: P-value for only high-dose BP switching. For all BP use, P = 0.164.

### 3.4 Hypercalcemia

Grade 3 hypercalcemia occurred in 3 patients, all after discontinuation. No grade 4 events were observed. All patients improved rapidly with treatment (zoledronic acid in 1, elcatonin in 1, and saline infusion in 1). Two cases occurred after SREs (6 days and 6 months later, respectively), and 1 occurred in a patient without SRE. No patients experienced both fragility fractures and grade ≥ 3 hypercalcemia.

All-grade hypercalcemia was observed in 14 patients during treatment and in 32 after discontinuation, representing a significant increase (McNemar’s chi-squared = 10.321, P = 0.0013). Patients with hypercalcemia before discontinuation showed a non-significant trend toward hypercalcemia after discontinuation (OR 3.16, 95 % CI 0.9–11.1, P = 0.072). During treatment, all cases were grade 1. After discontinuation, 1 patient developed grade 2 hypercalcemia and 3 developed grade 3 events. Among the 3 patients with grade 3 hypercalcemia, 2 had experienced grade 1 hypercalcemia 11 weeks earlier, while the other had normal calcium levels 8 days prior. Ten patients developed both hypercalcemia and SREs after discontinuation. Hypercalcemia preceded SREs in 7 patients (interval 4–319 days, mean 144 days), while SREs preceded hypercalcemia in 3 patients.

### 3.5 Serum ALP

**Fig. 2:**
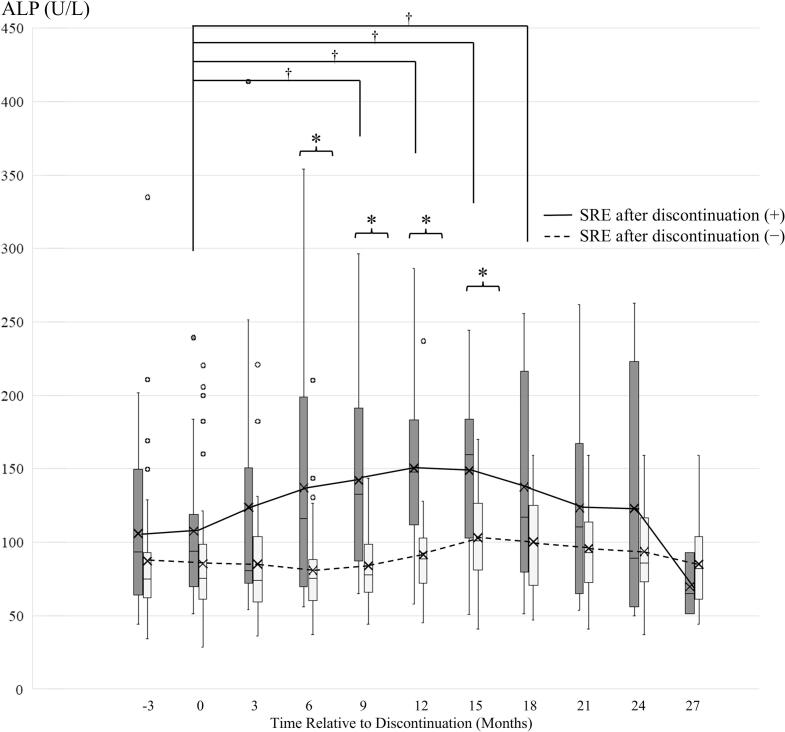
Longitudinal serum alkaline phosphatase (ALP) trajectories by post-discontinuation SRE status. Box-and-whisker plots of ALP at discontinuation, 3 months before, and every 3 months thereafter up to 27 months in patients with versus without SREs after discontinuation. Boxes represent the interquartile range, horizontal lines the median, whiskers extend to 1.5 × the interquartile range, and dots indicate outliers. A significant increase from the time of discontinuation (†) and a higher value compared to the group without SRE after discontinuation (*) were observed in the SRE group (P < 0.05, Wilcoxon tests for within-group comparisons, Mann–Whitney U tests for between-group comparisons). Solid and dashed lines indicate the average trend for each group.

**Fig. 3:**
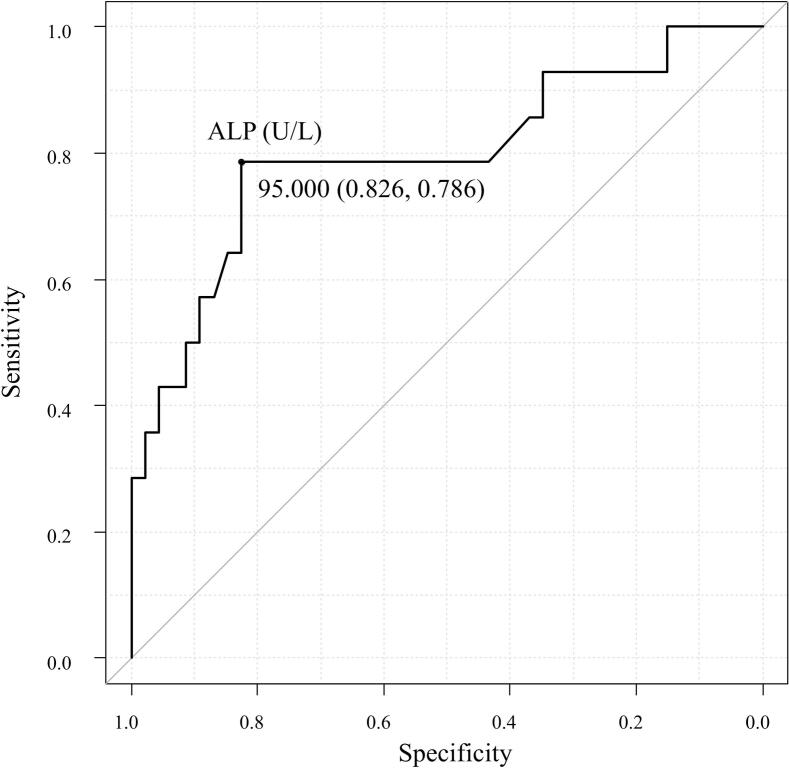
ROC curve of ALP after discontinuation for predicting subsequent SREs. Receiver-operating characteristic curve for ALP measured at approximately 6 months (±4 weeks) after discontinuation. The area under the curve (AUC) was 0.80 (95 % CI, 0.64–0.95). The Youden-optimized cutoff of ≥ 95 U/L yielded sensitivity 0.79 and specificity 0.83.

Fig. 2 shows ALP trends stratified by the presence of SREs after discontinuation. Twenty-four data points from 17 patients were excluded. At 6, 9, 12, and 15 months after discontinuation, ALP levels were significantly higher in patients with post-discontinuation SREs than in those without. In patients with SREs after discontinuation, ALP increased significantly from the time of discontinuation at 9, 12, 15, and 18 months. ROC analysis of ALP at 6 months after discontinuation for predicting subsequent SREs yielded a cutoff of ≥ 95 U/L, which maximized the sum of specificity (0.83) and sensitivity (0.79), with an area under the curve of 0.80 (95 % CI, 0.64–0.95) Fig. 3.

### 3.6 Benign fragility fractures

Benign fragility fractures occurred in 5 patients (6 events): 1 during treatment (spine), 1 within 4 weeks after discontinuation (femur), and 4 thereafter (spine 2, femur 1, and pubis 1). Neither of the two femoral fractures was an atypical femoral fracture. Three patients with benign fragility fractures also developed SREs after discontinuation. Four benign fragility fractures after discontinuation occurred at 7, 12, 14, and 30 months, respectively. Benign fragility fractures during treatment were 1.2 per 1000 person-month. After discontinuation, the incidence was 2.3 per 1000 person-months, representing a 2.0-fold increase, but not statistically significant (IRR 2.0, 95 % CI 0.4–10.6).

## 4 Discussion

This study is the first to demonstrate a transient increase in SREs after discontinuation of high-dose denosumab. The transient rise in SREs suggests that rebound activation of bone resorption following discontinuation may contribute to these events, underscoring the clinical risk of denosumab discontinuation. SREs significantly increased between 6 and 15 months after discontinuation. Furthermore, we observed that ALP levels transiently increased at 6, 9, and 12 months in patients who developed SREs after discontinuation. Although not statistically significant, the rise in SREs as early as 4 months after discontinuation, along with elevated ALP at 3 months in the SRE group, suggests that the risk of SREs may increase relatively early after discontinuation. This pattern appears to occur earlier than the rebound phenomenon typically reported with low-dose denosumab at around 6 months [11], and may represent a distinctive feature of high-dose discontinuation in cancer patients.

Although an increase in SREs after discontinuation of denosumab in cancer patients is anticipated, reports remain scarce [16], and the actual magnitude of risk has been unclear. Prior studies identified previous SREs and shorter treatment duration as risk factors for post-discontinuation SREs [16]. In the present analysis, using patients as their own controls, we demonstrated that SREs significantly increased 6–15 months after discontinuation, peaking at an 8.7-fold rise (95 %CI, 2.8–27.5) between 12 and 15 months. The decline in SRE incidence after 15 months contrasts with the expected progressive bone metastases over time. These findings suggest that the increase in SREs is transient and closely related to discontinuation of denosumab.

We examined whether SREs without progression of non-skeletal disease increased after discontinuation. Although the odds ratio was 2.33, the difference was not significant (P = 0.657). Adequate assessment of extra-skeletal progression was available for only 15 of 24 SREs, likely due to reduced imaging after disease progression, which may have limited statistical power. Notably, restricting the analysis to events after 6 months showed that 50 % occurred without non-skeletal progression, with an odds ratio of 5.0, underscoring the clinical relevance of rebound effects following discontinuation. While previous reports identified prior SREs as a risk factor for post-discontinuation SREs [16], we did not confirm this association. This may not be unexpected, as discontinuation of denosumab itself could contribute to an increased risk, independent of tumor progression.

In the analysis of the characteristics of SREs after discontinuation, a smaller number of denosumab doses was identified as a risk factor for post-discontinuation SREs, consistent with previous reports [16]. Interestingly, in osteoporosis treated with low-dose denosumab, greater numbers of doses have been associated with increased risk of rebound fractures [29], suggesting a contrasting phenomenon between high- and low-dose regimens. While deeper suppression of osteoclast activity is associated with stronger rebound, in the context of bone metastases, the control of tumor-related bone destruction may play a more critical role. Current ESMO guidelines recommend a minimum of 2 years of treatment [1].

Hypercalcemia is a major complication of bone metastases [30], but in cancer patients, it is often tumor-mediated and is usually excluded from recent analyses of SREs [[1], [2], [3]]. Nevertheless, rebound hypercalcemia after discontinuation has been sporadically reported to be symptomatic even in the absence of bone metastasis or cancer [1,21,22], and clinicians should remain alert to this risk. It is also of interest whether mild hypercalcemia could signal rebound bone resorption and thereby predict subsequent SREs. Although all-grade hypercalcemia significantly increased after discontinuation, its occurrence did not correlate with SRE development. In three of ten patients with both events, SREs preceded hypercalcemia. Because serum calcium levels are regulated by multiple factors, they may not directly reflect osteoclast activity and are therefore unlikely to serve as a reliable predictor of SREs. However, among patients without hypercalcemia during denosumab who later developed hypercalcemia after discontinuation, SREs were observed in 34.8 %, suggesting a possible association that warrants further study in larger cohorts. No associations were found between post-discontinuation SREs and sex, age, or pre-treatment hypercalcemia. Previous reviews have suggested that rebound hypercalcemia is more frequent in younger male patients and elderly women [21], but our findings suggest that baseline bone turnover may be less relevant for SRE risk.

ALP showed a transient increase, similar to the frequency of SREs, and elevated ALP (≥95 U/L) at 6 months after discontinuation may serve as a predictor of subsequent SREs. Whether this reflects rebound bone turnover or progression of bone lesions remains unclear and requires further investigation, as a slight baseline difference in ALP between patients with and without subsequent SREs was observed. Several studies have identified elevated ALP as a risk factor for SREs in patients with bone metastases, suggesting ALP serves as a marker of activated bone turnover [[23], [24], [25]]. Elevation of ALP may be a better predictor of SREs than serum calcium level. As ALP was not fractionated into *iso*-enzymes, more specific bone turnover markers including bone-specific alkaline phosphatase (BAP), procollagen type I N-terminal propeptide (P1NP), and tartrate-resistant acid phosphatase isoform 5b (TRACP-5b) should be evaluated in future studies.

This study also evaluated benign fragility fractures at non-metastatic sites. Benign fragility fractures occurred more frequently after discontinuation, which is consistent with low-dose denosumab [12,13]. However, this increase was not statistically significant. It is possible that in patients receiving high-dose denosumab, sufficient bone mass was accumulated so that, even when rebound bone resorption occurred, fragility fractures were temporarily prevented. In a trial of high-dose denosumab as adjuvant therapy in breast cancer, fractures occurred in 0.5 % of patients after treatment cessation [31]. Cancer patients are at high risk of treatment-induced bone loss (CTIBL) due to endocrine therapy, chemotherapy, glucocorticoid use, and other factors [[32], [33], [34]]. Thus, maintaining bone mass and preventing fractures are critical components of supportive care, regardless of bone metastases, and appropriate pharmacologic management—including careful attention to denosumab discontinuation—is also essential.

To prevent post-discontinuation SREs, no established strategies exist at present. Unintentional discontinuation due to missed orders or patient-requested discontinuation should be avoided. Even in cases of osteonecrosis of the jaw or invasive dental procedures, discontinuation of denosumab should be carefully considered [35]. If discontinuation is unavoidable, switching to bisphosphonates (BPs) may help mitigate rebound bone resorption [36]. However, in our analysis, post-discontinuation BP therapy was not associated with reduced SREs. Prior exposure to zoledronic acid before denosumab was also not associated with risk. Larger studies may yield different results; physicians need to follow ECTS recommendations [36] until more evidence of the preventive effect of BP becomes available.

Recent trials have considered extending the dosing interval of high-dose denosumab to 12 weeks to reduce adverse events such as osteonecrosis of the jaw [37]. Whether such regimens reduce rebound SREs after discontinuation is unknown. In our cohort, the mean dosing interval was 5.0 weeks, with only one case of intentional extension, precluding further analysis. Given that rebound occurs even with low-dose regimens, interval extension of high-dose denosumab may not completely prevent rebound SREs.

This study has several limitations. First, it was a single-center retrospective analysis with a relatively small sample size. Second, reasons for discontinuation were undocumented in many cases, introducing potential bias. Third, early SREs soon after treatment initiation and cases where SREs directly triggered discontinuation may have led to under- or overestimation of baseline incidence. Finally, because event numbers were low, incidence rates were estimated assuming a Poisson distribution, with multiple events per patient treated as independent. Although a sensitivity analysis using a generalized estimating equation (GEE) Poisson model yielded similar results, no correction for multiple comparisons was applied, necessitating cautious interpretation.

## 5 Conclusions

SREs significantly increased 6–15 months after discontinuation of high-dose denosumab. Elevated ALP during the same period was associated with increased risk of post-discontinuation SREs, and ALP value (≥95 U/L) at 6 months may be a useful predictor. These findings emphasize that discontinuation contributes to SRE risk possibly via rebound bone resorption. A smaller number of denosumab doses was also associated with a higher risk, emphasizing the danger of discontinuation before adequate disease control is achieved. Clinicians should recognize the risks associated with discontinuing denosumab and ensure its continuation whenever possible.

## Funding Statement

This research did not receive any specific grant from funding agencies in the public, commercial, or not-for-profit sectors.

## Ethics Statement

This study was approved by the Institutional Review Board of the authors’ institution (K2022037) and conducted in accordance with the Declaration of Helsinki.

## CRediT authorship contribution statement

**Nokitaka Setsu:** Writing – original draft, Project administration, Methodology, Formal analysis, Conceptualization. **Nobuhiko Yokoyama:** Methodology, Data curation. **Taito Esaki:** Writing – review & editing, Supervision. **Masafumi Yamaguchi:** Writing – review & editing, Data curation. **Eriko Tokunaga:** Writing – review & editing, Data curation. **Takahito Negishi:** Writing – review & editing, Data curation.

## Declaration of competing interest

The authors declare that they have no known competing financial interests or personal relationships that could have appeared to influence the work reported in this paper.

## References

[1] Coleman R., Hadji P., Body J.J., Santini D., Chow E., Terpos E., Oudard S., Bruland Ø., Flamen P., Kurth A., Van Poznak C., Aapro M., Jordan K. (2020). Bone health in cancer: ESMO Clinical Practice guidelines. Ann. Oncol..

[2] Stopeck A.T., Lipton A., Body J.J., Steger G.G., Tonkin K., de Boer R.H., Lichinitser M., Fujiwara Y., Yardley D.A., Viniegra M., Fan M., Jiang Q., Dansey R., Jun S., Braun A. (2010). Denosumab compared with zoledronic acid for the treatment of bone metastases in patients with advanced breast cancer: a randomized, double-blind study. J. Clin. Oncol..

[3] Henry D.H., Costa L., Goldwasser F., Hirsh V., Hungria V., Prausova J., Scagliotti G.V., Sleeboom H., Spencer A., Vadhan-Raj S., von Moos R., Willenbacher W., Woll P.J., Wang J., Jiang Q., Jun S., Dansey R., Yeh H. (2011). Randomized, double-blind study of denosumab versus zoledronic acid in the treatment of bone metastases in patients with advanced cancer (excluding breast and prostate cancer) or multiple myeloma. J. Clin. Oncol..

[4] von Moos R., Costa L., Gonzalez-Suarez E., Terpos E., Niepel D., Body J.J. (2019). Management of bone health in solid tumours: from bisphosphonates to a monoclonal antibody. Cancer Treat. Rev..

[5] Portenoy R.K., Lesage P. (1999). Management of cancer pain. Lancet.

[6] Roodman G.D. (2004). Mechanisms of bone metastasis. N. Engl. J. Med..

[7] Lipton A., Fizazi K., Stopeck A.T., Henry D.H., Brown J.E., Yardley D.A., Richardson G.E., Siena S., Maroto P., Clemens M., Bilynskyy B., Charu V., Beuzeboc P., Rader M., Viniegra M., Saad F., Ke C., Braun A., Jun S. (2012). Superiority of denosumab to zoledronic acid for prevention of skeletal-related events: a combined analysis of 3 pivotal, randomised, phase 3 trials. Eur. J. Cancer.

[8] Henry D., Vadhan-Raj S., Hirsh V., von Moos R., Hungria V., Costa L., Woll P.J., Scagliotti G., Smith G., Feng A., Jun S., Dansey R., Yeh H. (2014). Delaying skeletal-related events in a randomized phase 3 study of denosumab versus zoledronic acid in patients with advanced cancer: an analysis of data from patients with solid tumors. Support Care Cancer.

[9] Cadieux B., Coleman R., Jafarinasabian P., Lipton A., Orlowski R.Z., Saad F., Scagliotti G.V., Shimizu K., Stopeck A. (2022). Experience with denosumab (XGEVA®) for prevention of skeletal-related events in the 10 years after approval. J. Bone Oncol..

[10] Patrick D.L., Cleeland C.S., von Moos R., Fallowfield L., Wei R., Öhrling K., Qian Y. (2015). Pain outcomes in patients with bone metastases from advanced cancer: assessment and management with bone-targeting agents. Support Care Cancer.

[11] Bone H.G., Bolognese M.A., Yuen C.K., Kendler D.L., Miller P.D., Yang Y.C., Grazette L., San Martin J., Gallagher J.C. (2011). Effects of denosumab treatment and discontinuation on bone mineral density and bone turnover markers in postmenopausal women with low bone mass. J. Clin. Endocrinol. Metab..

[12] Cummings S.R., Ferrari S., Eastell R., Gilchrist N., Jensen J.B., McClung M., Roux C., Törring O., Valter I., Wang A.T., Brown J.P. (2018). Vertebral fractures after discontinuation of denosumab: a post hoc analysis of the randomized placebo-controlled FREEDOMtTrial and its extension. J. Bone Miner. Res..

[13] Anastasilakis A.D., Makras P., Yavropoulou M.P., Tabacco G., Naciu A.M., Palermo A. (2021). Denosumab Discontinuation and the Rebound Phenomenon: a Narrative Review. J. Clin. Med..

[14] Schini M., Gossiel F., Saini T., Banda P., Ward R., Vilaca T., Eastell R., Fontalis A. (2025). The effects of denosumab on osteoclast precursors in postmenopausal women: a possible explanation for the overshoot phenomenon after discontinuation. J. Bone Miner. Res..

[15] Kim A.S., Girgis C.M., McDonald M.M. (2022). Osteoclast recycling and the rebound phenomenon following denosumab discontinuation. Curr. Osteoporos. Rep..

[16] Jacobson D., Cadieux B., Higano C.S., Henry D.H., Bachmann B.A., Rehn M., Stopeck A.T., Saad H. (2022). Risk factors associated with skeletal-related events following discontinuation of denosumab treatment among patients with bone metastases from solid tumors: a real-world machine learning approach. J. Bone Oncol..

[17] Gossai N., Hilgers M.V., Polgreen L.E., Greengard E.G. (2015). Critical hypercalcemia following discontinuation of denosumab therapy for metastatic giant cell tumor of bone. Pediatr. Blood Cancer.

[18] Setsu N., Kobayashi E., Asano N., Yasui N., Kawamoto H., Kawai A., Horiuchi K. (2016). Severe hypercalcemia following denosumab treatment in a juvenile patient. J. Bone Miner. Metab..

[19] Roux S., Massicotte M.H., Huot Daneault A., Brazeau-Lamontagne L., Dufresne J. (2019). Acute hypercalcemia and excessive bone resorption following anti-RANKL withdrawal: Case report and brief literature review. Bone.

[20] Wang R., Renouf D.A. (2022). Rebound hypercalcemia post-denosumab cessation in metastatic breast cancer. Osteoporos Int..

[21] Horiuchi K., Kobayashi E., Mizuno T., Susa M., Chiba K. (2021). Hypercalcemia following discontinuation of denosumab therapy: a systematic review. Bone Rep..

[22] Villanova M., Chou S.H., Min L. (2025). Incidence of Hypercalcemia and Vertebral Fractures following Denosumab Withdrawal in Lung Cancer patients: a Longitudinal Cohort Study. J. Bone Metab..

[23] Miyashita H., Cruz C., Smith C. (2021). Risk factors of skeletal-related events in patients with bone metastasis from non-small cell lung cancer undergoing treatment with zoledronate-a post hoc analysis of a randomized clinical trial. Support Care Cancer.

[24] Miyashita H., Cruz C., Malamud S. (2020). Risk factors for skeletal-related events in patients with bone metastasis from breast cancer undergoing treatment with zoledronate. Breast Cancer Res. Treat..

[25] Y. Singh, S.K. Barua, S. Trivedi, R. Tp, M. Pratim Kashyap, L. Kumar Agrawal, U. Kumar Pathak, N. Garg, Skeletal-Related events in renal cell carcinoma: prediction with alkaline phosphatase (ALP), C-reactive Protein (CRP), Haemoglobin (Hb) and Erythrocyte Sedimentation Rate (ESR) (A.C.H.E.) Score for Risk Stratification, Cureus 15(6) (2023) e40835. 10.7759/cureus.40835 PMC10363263 37489216

[26] Hara K., Nagano N., Sato Y., Go H., Imaizumi T., Hijikata M., Fuwa K., Aoki R., Seimiya A., Okahashi A., Morioka I. (2025). Percentile-based reference values for serum alkaline phosphatase at birth in Japanese preterm and term infants: a retrospective cohort study. Medicine (Baltimore).

[27] Chen Q., Hu C., Liu Y., Song R., Zhu W., Zhao H., Nino A., Zhang F., Liu Y. (2018). Pharmacokinetics, pharmacodynamics, safety, and tolerability of single-dose denosumab in healthy chinese volunteers: a randomized, single-blind, placebo-controlled study. PLoS One.

[28] Kanda Y. (2013). Investigation of the freely available easy-to-use software 'EZR' for medical statistics. Bone Marrow Transplant..

[29] Cosman F., Huang S., McDermott M., Cummings S.R. (2022). Multiple vertebral fractures after denosumab discontinuation: FREEDOM and FREEDOM extension trials additional post hoc analyses. J. Bone Miner. Res..

[30] Skeletal Complications of Malignancy. Proceedings of a symposium. Bethesda, Maryland, April 19-20, 1997, Cancer 80(8 Suppl) (1997) 1527-701. 9377495

[31] Coleman R., Finkelstein D.M., Barrios C., Martin M., Iwata H., Hegg R., Glaspy J., Periañez A.M., Tonkin K., Deleu I., Sohn J., Crown J., Delaloge S., Dai T., Zhou Y., Jandial D., Chan A. (2020). Adjuvant denosumab in early breast cancer (D-CARE): an international, multicentre, randomised, controlled, phase 3 trial. Lancet Oncol..

[32] D'Oronzo S., Stucci S., Tucci M., Silvestris F. (2015). Cancer treatment-induced bone loss (CTIBL): pathogenesis and clinical implications. Cancer Treat. Rev..

[33] J.R. Gralow, J.S. Biermann, A. Farooki, M.N. Fornier, R.F. Gagel, R. Kumar, G. Litsas, R. McKay, D.A. Podoloff, S. Srinivas, C.H. Van Poznak, NCCN task force report: bone health in cancer care, J. Natl. Compr. Canc. Netw. 11 Suppl 3 (2013) S1-50; quiz S51. 10.6004/jnccn.2013.0215 23997241

[34] Hadji P., Aapro M., Al-Dagri N., Alokail M., Biver E., Body J.J., Brandi M.L., Brown J., Confavreux C., Cortet B., Drake M., Ebeling P., Eriksen E.F., Fuleihan G.E., Guise T.A., Harvey N.C., Kurth A., Langdahl B., Lems W., Matijevic R., McCloskey E., Nappi R., Palacios S., Pfeiler G., Reginster J.Y., Rizzoli R., Santini D., Tuzun S., Poznak C.V., Villiers T., Zillikens M.C., Coleman R. (2025). Management of aromatase inhibitor-associated bone loss (AIBL) in women with hormone-sensitive breast cancer: an updated joint position statement of the IOFCABS, ECTS, IEG, ESCEO, IMS, and SIOG. J Bone Oncol.

[35] Ruggiero S.L., Dodson T.B., Aghaloo T., Carlson E.R., Ward B.B., Kademani D. (2022). American Association of Oral and Maxillofacial Surgeons' Position Paper on Medication-Related Osteonecrosis of the Jaws-2022 Update. J. Oral Maxillofac. Surg..

[36] Tsourdi E., Zillikens M.C., Meier C., Body J.J., Gonzalez Rodriguez E., Anastasilakis A.D., Abrahamsen B., McCloskey E., Hofbauer L.C., Guañabens N., Obermayer-Pietsch B., Ralston S.H., Eastell R., Pepe J., Palermo A., Langdahl B. (2020). Fracture risk and management of discontinuation of denosumab therapy: a systematic review and position statement by ECTS. J. Clin. Endocrinol. Metab..

[37] Keisner S.V. (2024). Prevention of skeletal-related events with extended-interval denosumab: a review of the literature. Ann. Pharmacother..

