# Supplementary figures and images for: Transient increase in skeletal-related events after discontinuation of high-dose denosumab in cancer patients
Source: J Bone Oncol. 2025 Oct 11;55:100717. doi: 10.1016/j.jbo.2025.100717 (PMC12547457; doi:10.1016/j.jbo.2025.100717)

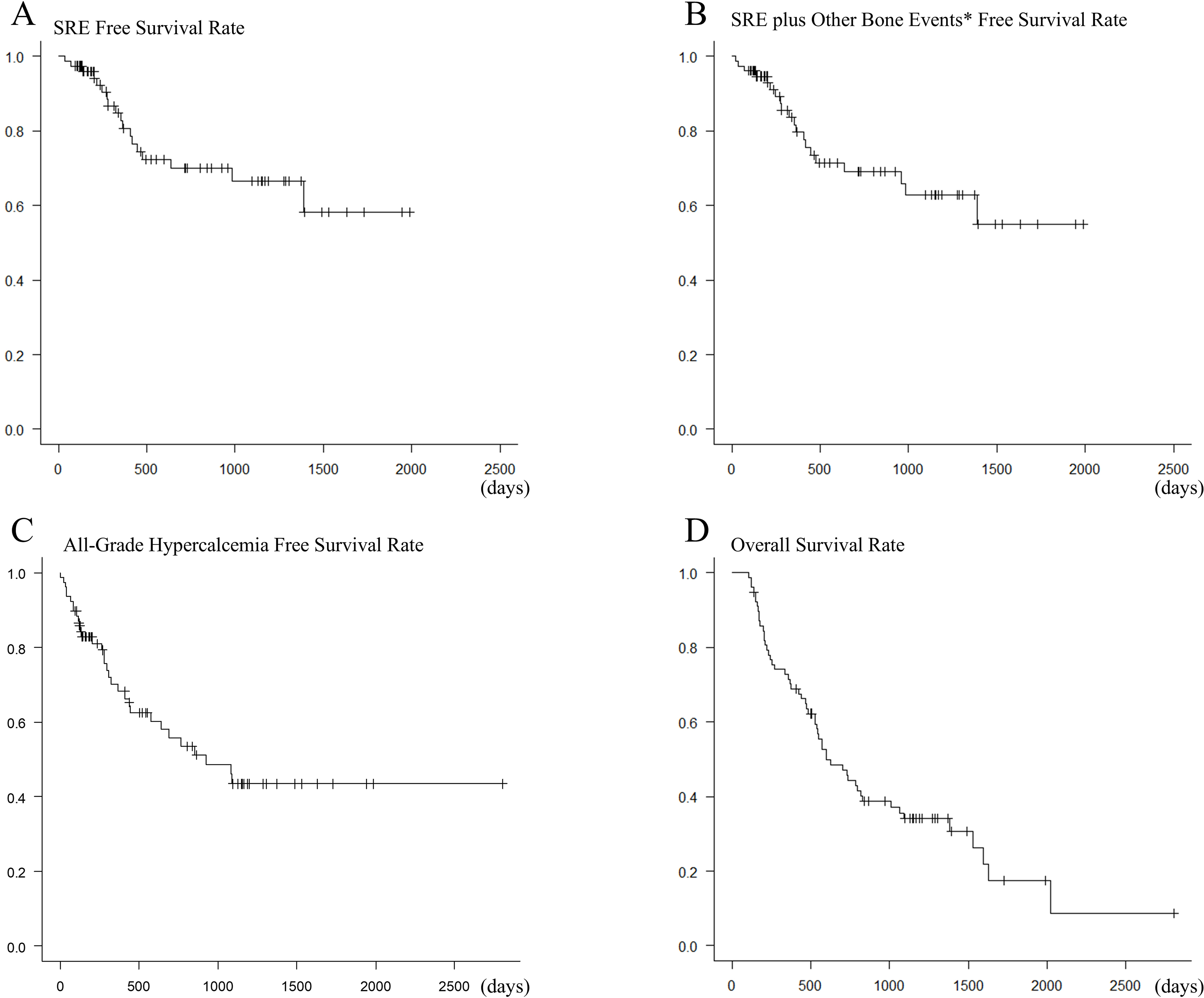

Supplement: Supplementary Fig. 1 [file mmc1.jpg]

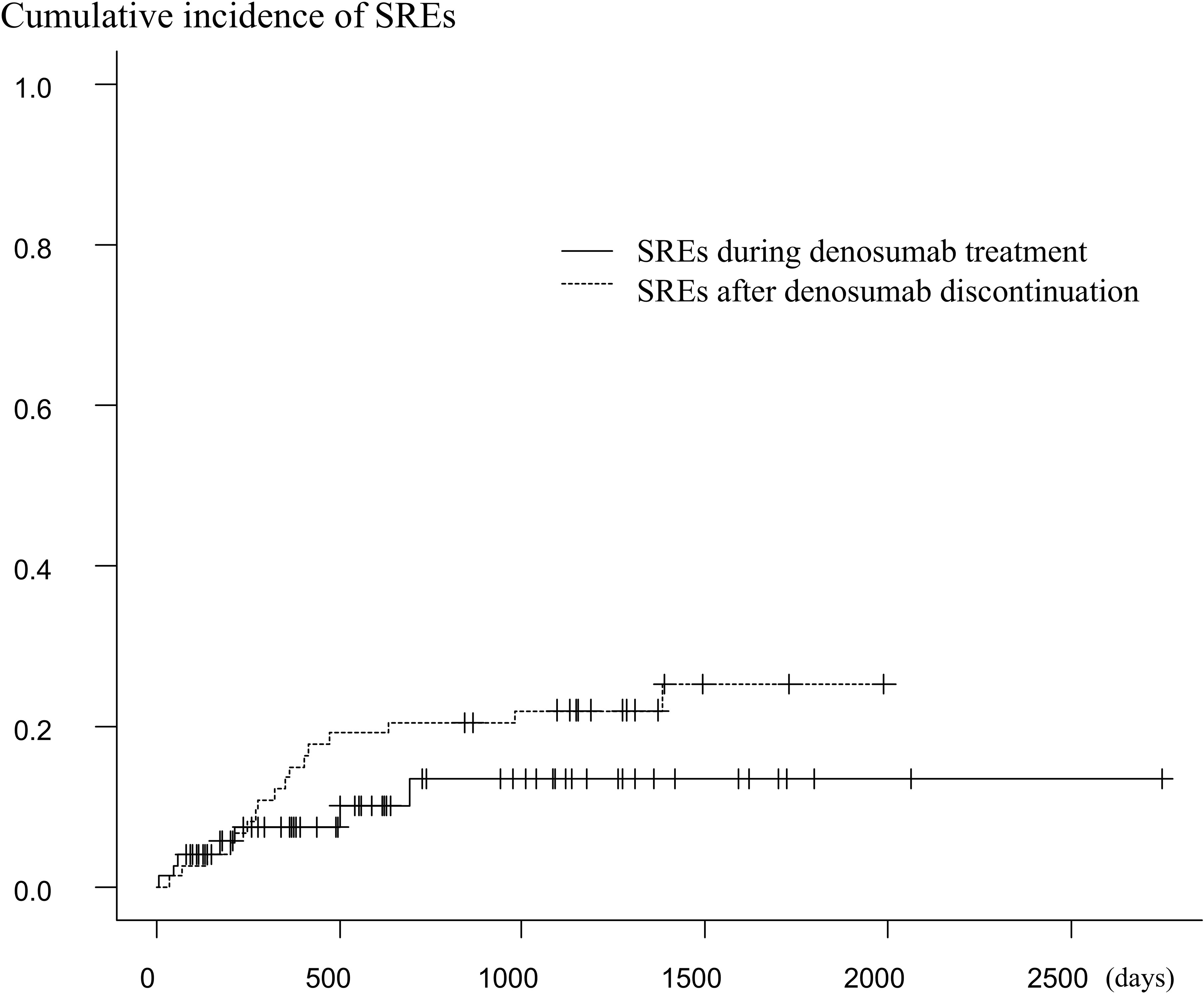

Supplement: Supplementary Fig. 2 [file mmc2.jpg]
